# Adeno-Associated virus-based approaches for mitochondrial diseases: advances and challenges

**DOI:** 10.1038/s41380-026-03570-y

**Published:** 2026-04-29

**Authors:** Samantha Corrà, Valeria Balmaceda, Carlo Viscomi

**Affiliations:** 1 Veneto Institute of Molecular Medicine (VIMM), Via Orus, 2-35129, Padova, Italy; 2 Department of Biomedical Sciences, University of Padova, Via Ugo Bassi, 58/B-35131, Padova, Italy; 3 Department of Biosciences, University of Milano, via Celoria 26-20133, Milano, Italy

**Keywords:** Genetics, Psychiatric disorders

## Abstract

Mitochondrial diseases, caused by mutations in either mitochondrial or nuclear DNA, are highly complex genetic disorders characterized by faulty oxidative phosphorylation. Adeno-associated virus (AAV)-based gene therapy with its broad and customizable tissue tropism achieved through natural and engineered serotypes offers a highly effective platform for delivering therapeutic genes to affected tissues. However, the intricate genetics and biology of mitochondria present unique challenges for the development of AAV-based therapies. While gene replacement therapy remains a viable strategy for correcting nuclear gene defects, mutations in mtDNA require specialized approaches, such as mitochondrially targeted, RNA-free base editors and nucleases capable of precise editing within the mitochondrial genome. As an alternative, allotopic expression, which involves expressing mitochondrial genes from the nuclear genome, is currently being evaluated in clinical trials but remains controversial, due to issues related to mitochondrial import and functional integration in the respiratory complexes. The clinical translation of AAV-mediated therapies for mitochondrial diseases still confronts several interrelated challenges, including efficient targeting of multiple affected organs, scalable and cost-effective vector manufacturing, and minimizing vector-associated toxicity. By integrating advanced genome editing technologies with sophisticated vector engineering and delivery strategies, AAV-based gene therapy stands as a transformative approach for addressing the broad and heterogeneous spectrum of primary mitochondrial disorders. Continued progress in overcoming current biological and technical barriers will be essential to realize the full therapeutic potential of AAVs.

## Introduction

**Fig. 1:**
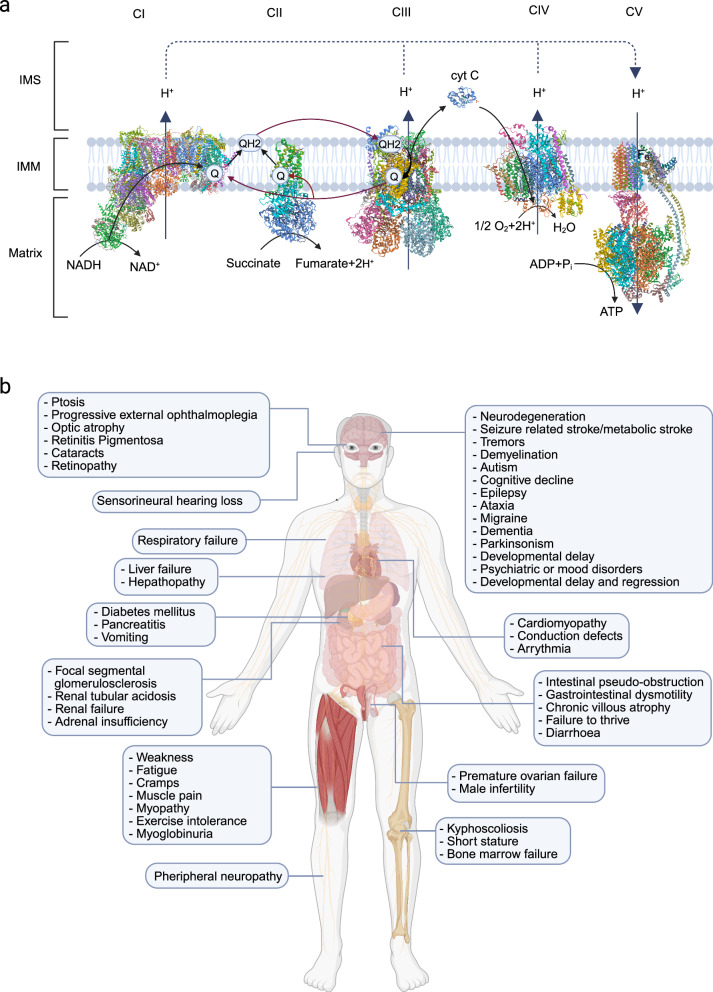
Electron transport system and mitochondrial diseases. **a** *Oxidative phosphorylation scheme*. The process involves a series of protein complexes located in the inner mitochondrial membrane (IMM), specifically, complexes I to IV of the electron transport chain, and complex V or ATP synthase. Complex I (NADH:coenzyme Q oxidoreductase) carires out the transfer of electrons from NADH to coenzyme Q10 (CoQ), reducing it from its oxidized form (ubiquinone) to its reduced form (ubiquinol, QH₂), while contributing to the formation of a proton gradient. Complex II (succinate-CoQ oxidoreductase) bridges the TCA cycle and the electron transport chain by oxidizing succinate and reducing CoQ to QH₂. Complex III (ubiquinol-cytochrome c oxidoreductase) transfers electrons from ubiquinol to cytochrome c, continuing the buildup of the proton gradient across the membrane. Complex IV (cytochrome c oxidase) transfers electrons to molecular oxygen, forming water and further contributing to the proton motive force. Finally, complex V (ATP synthase) uses the energy stored in the transmembrane proton gradient to drive the synthesis of ATP from ADP and inorganic phosphate through a mechanical rotation mechanism. OMM: outer mitochondrial membrane; IMS: intermembrane space; IMM: inner mitochondrial membrane; e^-^: electron; P: phosphate; CoQ: coenzyme Q; Cyt c: cytochrome c. **b** *Clinical spectrum of mitochondrial diseases*. Mitochondrial disorders present with diverse symptoms, that can affect any organ or tissue. Clinical features are typically multisystemic and may be broadly categorized into neurological and non-neurological manifestations, with significant variability between individuals. Figure prepared with Biorender.

Mitochondria are essential organelles responsible for converting the energy derived from nutrients into adenosine triphosphate (ATP), a high-energy molecule readily used by the cells, through a process known as oxidative phosphorylation (OXPHOS; Fig. 1) [1]. This intricate process occurs within the inner mitochondrial membrane (IMM), where four multi-protein complexes (Complexes I–IV, CI-IV) and the mitochondrial ATP synthase (Complex V, CV), along with the mobile electron carriers cytochrome c and coenzyme Q, coordinate the transfer of electrons to molecular oxygen through the electron transport system (ETS) [2]. As electrons move through the ETS, protons are pumped from the mitochondrial matrix into the intermembrane space, establishing an electrochemical gradient (Dy) which is then harnessed by ATP synthase to generate ATP from ADP and inorganic phosphate [3, 4]. Beyond energy conversion, mitochondria play central roles in numerous other cellular functions, including apoptosis, calcium buffering, redox balance, and the biosynthesis of essential prosthetic groups for several proteins, such as heme, steroids, and iron-sulfur clusters. These diverse functions are critically dependent on the integrity of the OXPHOS system, making it a key node of cellular physiology [5].

Defects in OXPHOS can arise from mutations in either mitochondrial DNA (mtDNA) or nuclear DNA (nDNA), reflecting the dual-genomic origin of the mitochondrial proteome [6]. The mitochondrial genome is a small circular DNA which is present in hundreds to thousands of copies in each cell. The multicopy nature of mtDNA implies that mutations arising in it only affect part of the molecules (a condition known as heteroplasmy) and remain silent until they exceed a certain threshold [7–9]. This has important implications for the therapy, as interventions aimed at reducing the mutational burden below the threshold may be beneficial to the patients. It should be noted, however, that also homoplasmic mutations, i.e. in which all the mtDNA molecules are mutated, may cause mitochondrial diseases. MtDNA encodes 13 core subunits of the ETS, along with 22 tRNAs and 2 rRNAs required for mitochondrial protein translation. The vast majority of the mitochondrial proteome is encoded by nDNA, synthesized in the cytoplasm, and imported into mitochondria through an ATP-dependent mechanism [10, 11].

Pathogenetic mutations affecting any component of this system can impair the assembly or function of OXPHOS complexes, leading to a spectrum of mitochondrial diseases. These disorders often present with multisystem involvement, affecting tissues with high energy demands such as the brain, heart, and skeletal muscle, although any other organ can be affected (Fig. 1b). Clinically, they are associated with extremely heterogeneous multisystem syndromes, such as Leigh disease, mitochondrial encephalomyopathy, lactic acidosis, and stroke-like episodes (MELAS), myoclonic epilepsy with ragged-red fibres (MERRF), Leber’s hereditary optic neuropathy (LHON), and many others [7, 12–16]. The clinical manifestations of mitochondrial disorders are influenced not only by the nature of the underlying genetic defect but also by the heteroplasmy levels (at least for mtDNA-related diseases), the interactions with the environment and genetic and epigenetic modifiers.

The biochemical, genetic, and clinical heterogeneity of mitochondrial diseases poses major challenges for diagnosis, disease modelling, and treatment development. Despite major advances in next-generation sequencing technologies, including the emergence of third-generation, long-read platforms [17–21], that have greatly expanded our ability to detect genetic variants, many patients still lack a definitive molecular diagnosis [22], and therapeutic options remain largely supportive rather than curative.

In recent years, gene therapy has emerged as a promising avenue to etiologically correct mitochondrial dysfunction [23]. Among the various delivery platforms, adeno-associated viruses (AAVs) have gained significant attention due to their favourable safety profile, broad tropism, and ability to mediate long-term gene expression in post-mitotic tissues [24–26]. AAV-based approaches offer the potential to correct mitochondrial defects due to mutations in nDNA. Rapid technological innovations now allow the manipulation of the mitochondrial genome as well. However, the development of effective AAV-based therapies for OXPHOS defects faces substantial hurdles, including limitations in packaging capacity, immune responses, and the unique challenges of delivering functional proteins inside mitochondria. An additional challenge stems from the fact that mitochondria have a dedicated transcriptional and translational apparatus and employs a genetic code, which differs from the universal one. This review aims to explore the current landscape of AAV-based gene therapy strategies to rescue OXPHOS defects, to highlight recent advances, and to critically evaluate the obstacles that remain in translating these approaches from bench to bedside.

## AAV-based gene therapy for mitochondrial diseases

### AAVs as therapeutic tools

**Fig. 2:**
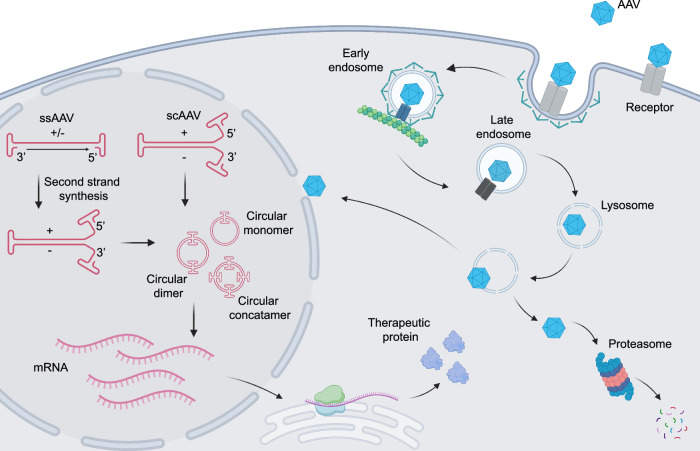
Mechanism of AAV vector transduction. *Mechanism of AAV vector transduction*. Adeno-associated virus (AAV) binds to glycosylated cell surface receptors, initiating clathrin-mediated endocytosis and internalization into endosomes. After endosomal escape, AAV particles may either be ubiquitylated for proteasomal degradation or trafficked to the nucleus. Following endosomal escape, AAV undergoes transport into the nucleus and uncoating. AAV can also undergo proteolysis by the proteasome. There are currently two classes of recombinant AAVs (rAAVs) in use: single-stranded AAV (ssAAV) and self-complementary AAV (scAAV). ssAAVs are packaged as either sense (plus-stranded) or anti-sense (minus-stranded) genomes. These single-stranded forms are still transcriptionally inert when they reach the nucleus and must be converted to double-stranded DNA as a prerequisite for transcription. This conversion can be achieved by second strand synthesis via host cell DNA polymerases or by strand annealing of the plus and minus strands that may coexist in the nucleus. Because scAAVs are already double stranded by design, they can immediately undergo transcription. The viral inverted terminal repeats (ITRs) present in the rAAV genome can drive inter-molecular or intra-molecular recombination to form circularized episomal genomes that can persist in the nucleus. ssAAV, single-stranded AAV; scAAV: self-complementary AAV. Figure prepared with Biorender.

AAV vectors are currently the most widely used system for gene therapy, owing to their unique combination of safety, efficiency, and versatility. Hundreds of clinical trials are currently ongoing using AAVs on different conditions, including many genetic diseases (https://clinicaltrials.gov/search?term=AAV). AAV is a small, non-enveloped virus with a single-stranded DNA genome belonging to the *Parvoviridae* family [27, 28] (Fig. 2). It naturally infects humans and certain non-human primates and is not associated with any known pathology [29–31] making it an attractive platform for clinical applications. One of the most notable advantages of AAV vectors is their capacity to mediate long-term transgene expression in post-mitotic cells such as neurons, cardiomyocytes, and skeletal muscle fibres, that are critically involved in many mitochondrial and OXPHOS-related disorders.

AAV vectors can be engineered to carry therapeutic transgenes by removing the viral coding sequences and replacing them with a gene of interest, flanked by inverted terminal repeats (ITRs) necessary for replication and packaging [24]. Although its packaging capacity is limited to approximately 4.7 kilobases, AAVs broad tissue tropism, which can be further refined using engineered capsids, allows for targeted delivery to specific organs or cell types. This tropism can be further fine-tuned through the use of tissue-specific promoters and regulatory elements, enhancing both precision and efficacy of gene delivery. Traditionally, strong constitutive promoters, such as CMV, CBA, EF1α, and PGK, have been widely used due to their ability to drive robust and long-lasting expression across various tissues. However, these promoters are associated with several potential drawbacks, including susceptibility to transcriptional silencing, immune activation, off-target expression, and potential toxicity. The use of tissue-specific promoters offers improved safety and therapeutic precision by restricting transgene expression to target cells. While these promoters generally produce lower expression levels than constitutive promoters, their activity can be significantly enhanced through the addition of upstream enhancer elements or by employing emerging strategies such as CRISPR-based transcriptional activation [32–34].

Notably, self-complementary (sc)AAVs, which contain an inverted repeat genome folded into dsDNA, have been engineered to bypass the rate limiting step of the second DNA strand synthesis, thus allowing for a quicker expression of the therapeutic gene. However, as a consequence of this particular DNA structure, scAAVs have a reduced cloning capacity compared to single-stranded vectors [35] (Fig. 2).

AAV vectors exhibit low immunogenicity compared to other viral platforms and primarily persist as episomal DNA in the nucleus, minimizing the risk of insertional mutagenesis, a key concern in integrating viral vectors, and are thus considered overall safe. A recurrent site of integration has been reported in chromosome 19 (AAVS1) [36], and the integration of therapeutic AAVs has been reported, but not associated with development of tumours [37]. AAV vectors have already demonstrated clinical success in treating monogenic disorders, such as spinal muscular atrophy (SMA) (NCT03306277) and Leber’s congenital amaurosis (LCA)(NCT02781480), providing strong proof-of-concept for broader applications in other human genetic disorders.

Another promising strategy to address the limitations of naturally occurring AAVs is the use of pseudotyped vectors, produced by cross-packaging the genome of one parvovirus within the capsid of another. This approach yields a chimeric vector designed to unite the key advantages of each component, for example, pairing the low genotoxicity of an episomal AAV genome with the distinct tissue tropism and/or expanded cargo capacity provided by the heterologous parvovirus capsid. Yan et al. packaged an AAV genome within a human bocavirus 1 (HBoV1) capsid, creating AAV/HBoV1 chimeric vectors that efficiently and selectively transduced polarized human airway epithelia. This strategy is particularly compelling because wild-type HBoV1 has a larger 5.5-kb genome compared with the capacity of most AAV serotypes [38].

These features collectively make AAVs a compelling vehicle to deliver therapeutic genes and reaching an etiological correction or compensation of OXPHOS defects. However, several technical and translational challenges remain, including pre-existing neutralizing antibodies against common AAV serotypes, the limited cargo capacity, and the current inability to efficiently deliver genetic material directly into mitochondria. Overcoming these obstacles will require continued innovation in capsid engineering, vector design, and delivery strategies.

### AAV therapy for mitochondrial diseases due to mutations in nuclear genes

Mitochondrial disorders frequently arise from mutations in nuclear genes encoding core components or assembly factors of the OXPHOS system, mitochondrial DNA replication and maintenance machinery, or factors required for mitochondrial protein translation [39]. AAV-mediated gene therapy has demonstrated strong therapeutic efficacy in correcting mutations within nuclear-encoded mitochondrial genes (Table 1) [40].

**Table 1:** Experimental gene therapy in mouse models of defects of complex I and IV defects.

AAV-BASED GENE THERAPY	GENE	HUMAN PHENOTYPE	SEROTYPE	ROUTE	TARGET	REFERENCES
Complex I	*NDUFS3*	Leigh syndrome	AAV9	i.v.	Skeletal muscle	[43]
			AAV-PHP.eB	i.v.	CNS	[45]
	*NDUFS4*	Leigh syndrome	AAV-PHP.B	i.v.	CNS	[49]
			AAV9	i.v.+i.c.v.	CNS and peripheral tissues	[50]
			scAAV9	i.c.v. + i.v.	CNS and peripheral tissues	[51]
Complex IV	*COX10*	Leigh syndrome Encephalopathies Hypertrophic cardiomyopathy	AAV-PHP.eB	i.v.	CNS	[45]
	*SURF1*	Leigh syndrome	scAAV9	i.t.	Brain, liver, and muscle	[55]
	*ETHE1*	Encephalopathy Myopathy Systemic vascular dysfunction	AAV8	i.c.	Liver	[58]

#### AAV-based gene therapy for complex I and IV subunits and assembly factors

Complex I (NADH:ubiquinone oxidoreductase) [41] and Complex IV (cytochrome c oxidase) [42] deficiencies are among the most prevalent and clinically severe subtypes of mitochondrial disease, frequently associated with early-onset encephalopathies, such as Leigh syndrome. These multi-subunit complexes are composed of both mitochondria and nucleus-encoded proteins. Several studies have demonstrated that AAV-mediated delivery of nuclear-encoded mitochondrial genes can restore bioenergetic proficiency and improve survival in mouse models of Complex I and IV deficiency (Table 1).

In a skeletal muscle–specific *Ndufs3*^*-/-*^ model, systemic delivery of AAV9-*Ndufs3* effectively prevented and reversed myopathy [43]. Early intervention restored NDUFS3 expression and delayed disease onset, while post-symptomatic intervention improved mitochondrial function and muscle strength, even after prolonged complex I deficiency, highlighting both the therapeutic potential and metabolic resilience and plasticity of skeletal muscle.

Expanding on this, Walker et al. employed CNS-specific *Ndufs3*^*-/-*^ and *Cox10*^*-/-*^ models to show that retro-orbital delivery of AAV-PHP.eB, an engineered derivative of AAV9 characterized by a remarkable capacity to cross the blood-brain barrier (BBB) [44], to express the missing genes, significantly prolonging survival from 5–6 months to over 15 months, despite transducing only ~30% of neurons. Treated mice displayed near-complete correction of disease phenotypes and restoration of key cellular and metabolic parameters, emphasizing the potential for partial gene rescue to yield full functional benefit [45].

Additional evidence for the feasibility of AAV therapy in Complex I deficiency comes from *Ndufs4*^*-/-*^ models, which recapitulate the neuropathological and metabolic features of Leigh syndrome [46, 47]. Treatment with AAV-PHP.B, which has high capacity to cross the BBB similar to PHP.eB [44], and AAV9 vectors encoding the human *NDUFS4* extended lifespan and improved neuromotor function [48, 51]. A single administration of AAV-PHP.B-*hNDUFS4* at one month of age was shown to slow disease progression improving lifespan to 250 days in 50% of the treated animals [48–50]. However, this engineered AAV9 variant, is only effective in some mouse strains and cannot be used in primates because they lack the specific receptor mediating the extraordinary capacity of PHP.B to cross the BBB [52, 53]. Combined intravenous (IV) and intracerebroventricular (ICV) administration of scAAV9 in neonatal mice resulted in restored complex I activity in both the CNS and peripheral tissues, improved coordination, and prolonged survival—demonstrating that addressing both central nervous system and peripheral organ involvement is essential for effectively managing [51]

In another experimental setting, AAV-h*SURF1*, expressing a CIV assembly factor, has been investigated in *Surf1*^*-/-*^ mice [54]. Ling et al. showed that intrathecal (IT) administration of scAAV9- h*SURF1* under the control of the hybrid chicken β-actin (CBA) promoter, alone or combined with IV administration, produced modest yet statistically significant increases in complex IV activity across brain, liver, and muscle. Although exercise-induced lactate elevations were attenuated post-treatment, indicating some degree of functional rescue, overall clinical benefit remained limited [55]. However, the *Surf1*^*-/-*^ mouse does not fully recapitulate the human Leigh syndrome phenotype, which complicates interpretation of translational relevance.

Ethylmalonic encephalopathy (EE), caused by *ETHE1* mutations, leads to accumulation of H₂S, a gasotransmitter which strongly inhibits cytochrome c oxidase at high doses, leading to encephalopathy, myopathy and systemic vascular dysfunction [56, 57]. The *Ethe1*^*-/-*^ mouse model recapitulates the key features of the human disease. Liver-specific re-expression of h*ETHE1* in the mouse model of the disease by using the hepatotropic AAV8 with the liver-specific TBG promoter resulted in full restoration of the sulfide oxidase enzymatic activity encoded by *ETHE1*, extended the lifespan from ~26 to over 180 days in knockout mice, and markedly reduced the biomarkers of the disease [58]. These findings support liver-directed therapy as a practical and minimally invasive treatment strategy for metabolic mitochondrial diseases characterized by the accumulation of toxic compounds [59]

#### AAV-based gene therapy for factors involved in mtDNA maintenance

Mitochondrial DNA depletion syndromes (MDDS) encompass a heterogeneous group of early-onset disorders characterized by a marked reduction in mtDNA copy number, leading to defective oxidative phosphorylation and severe multi-organ pathology. These are mostly due to mutations in genes encoding for factors directly involved in mtDNA replication and nucleotide supply. Additional mutations causing mtDNA instability syndromes have been found in genes involved in mitophagy, mitodynamics and in poorly characterized genes [60]. A number of preclinical experiments have been carried out in mouse models of MDDS (Table 2).

**Table 2:** Experimental gene therapy in mouse models of defects of MDDS and other mitochondrial diseases.

GENE	HUMAN PHENOTYPE	SEROTYPE	ROUTE	TARGET	REFERENCES
*MPV17*	Hepatocerebral disease.	AAV8	i.v.	Liver	[67]
*DGUOK*	Hepatocerebral disease.	AAV9	i.v.	Liver	
*TYMP*	Neurogastrointestinal encephalomyopathy.	AAV8	i.v.	Liver	[72, 73]
*TK2*	Myopathy.	AAV9	i.v.	Liver, CNS and skeletal muscles	[78]
*Ant1*	Myopathy. Progressive external ophthalmoplegia.	AAV2	i.m.	Skeletal muscle	[80]
*OPA1*	Dominant Optic Atrophy.	AAV2	intravitreous	Eye	[84]
*AIFM1*	Progressive optic nerve degeneration.	AAV2	intravitreous	Eye	[86]
*FXN*	Friedreich’s ataxia.	AAVrh10	i.v.	Heart and skeletal muscles	[91]
			i.v.	Heart	[92]
			i.v.	CNS	[93]
		AAV9	i.v.	CNS	[93]
*TAZ*	Barth syndrome.	AAV9	i.v.	Heart and skeletal muscle	[98]
			i.v.; s.c	Heart	[99]
*SLC25A46*	Neurodegeneration.	AAV-PHP.B	i.v.	CNS	[101]

Among these, MPV17-related hepatocerebral MDDS presents in infancy with hypoglycemia, hepatopathy, and progressive liver failure related to profound mtDNA depletion in the liver, and to a lesser extent, skeletal muscle [61], although other phenotypes have been described, including multisystem disorder with multiple mtDNA deletions, neuropathy with leukoencephalopathy and axonal polyneuropathy [61–64]. *MPV17* encodes a small hydrophobic protein of the inner mitochondrial membrane, possibly acting as a carrier of nucleotides [65]. Systemic administration of an AAV8 vector encoding human *MPV17* under a hepatocyte-specific thyroxine-binding globulin (TBG) promoter in *Mpv17*-knockout mice [66], successfully restored mtDNA content in liver, normalized serum transaminases and the activities of respiratory complexes. Notably, early (pre-symptomatic) intervention prevented the onset of liver fibrosis and cirrhosis, whereas post-symptomatic treatment stabilized pathology but could not reverse advanced hepatic damage due to dilution of the therapeutic gene due to the proliferation of the hepatocytes upon liver damage [67].

Mutations in *DGUOK*, encoding the deoxyguanosine kinase, a key enzyme in the mitochondrial nucleotide salvage pathway responsible for phosphorylating deoxyguanosine and deoxyadenosine, are another prominent cause of hepatocerebral MDDS, accounting for up to 20% of the cases [68, 69]. Keshavan et al. used AAV9 to express *hDGUOK* in a neonatal *Dguok*-knockout mouse model. The treatment led to robust, dose-dependent transduction and long-term hepatic expression of the transgene. Importantly, mtDNA copy number was restored, activities of ETS complexes I, III, and IV improved, and hepatocellular injury markers were significantly reduced. Survival was extended in a dose-responsive manner, confirming the feasibility of postnatal gene therapy for DGUOK-related disease when administered at an early developmental stage [70].

Mitochondrial neurogastrointestinal encephalomyopathy (MNGIE) is caused by loss-of-function mutations in *TYMP*, leading to systemic thymidine and deoxyuridine accumulation, impaired mtDNA replication, and multiple mtDNA deletions [71]. Early lentiviral ex vivo gene therapy followed by hematopoietic stem cell transplantation, corrected the biochemical defects [72] but raised integration-related safety concerns, shifting efforts toward AAV-based liver-directed strategies. An AAV8 approach using the TBG promoter achieved long-term metabolic correction in mice [73, 74]. Subsequent studies identified the alpha-1-antitrypsin promoter as more effective for *TYMP* expression [75]. Recent work has explored CRISPR-Cas9-mediated knock-in of *hTYMP* at safe genomic loci, by using either AAV8 or lipid nanoparticle–delivered Cas9 mRNA, resulting in durable expression and robust biochemical rescue [76].

Another mtDNA depletion syndrome, TK2 deficiency, causes severe mitochondrial myopathy due to defective phosphorylation of pyrimidine deoxynucleosides [77]. AAV9-mediated TK2 gene delivery in knock-in mice restored enzyme activity, normalized mtDNA content, and extended survival; co-administration of deoxycytidine and thymidine further enhanced outcomes [78].

Mutations in the ANT1 gene, which encodes the adenine nucleotide translocator essential for exporting ATP from mitochondria in exchange for ADP, cause mtDNA instability and mitochondrial myopathy [79]. AAV-mediated expression of *Ant1* restored ANT1 protein levels, rescued biochemical deficits and improved muscle structure in knockout mice [80].

#### AAV-based gene therapy for other causes of mitochondrial disease

AAVs have been used also in several other models of other mitochondrial diseases (Table 2). Dominant Optic Atrophy (DOA), caused primarily by haploinsufficiency of *OPA1*, a key regulator of mitochondrial fusion and cristae remodelling, represents a compelling target for gene therapy [81, 82]. Until recently, AAV-based approaches have been limited to LHON, with DOA receiving little attention [83]. In a preclinical study using a DOA mouse model carrying the common c.2708_2711delTTAG *OPA1* mutation, AAV-mediated expression of wild-type human *OPA1* under a CMV promoter effectively preserved retinal ganglion cells (RGCs) and prevented optic nerve degeneration, the pathological hallmark of DOA [84].

Apoptosis-Inducing Factor 1 (AIFM1) deficiency, as modelled in the *Harlequin* (Hq) mouse [85], results in complex I deficiency and progressive optic nerve degeneration. Intravitreal administration of AAV2 expressing full-length *Aifm1* preserved RGCs, restored ETS activities, and maintained visual function [86]. In a follow-up study using a late-stage disease model, both AAV-mediated *Aifm1* and *neuroglobin (Ngb)* intravitreal administration improved visual cortex responses, reduced gliosis, and preserved mitochondrial morphology even after significant RGC loss [87]. Stereotactic injections of AAV2/9 vectors encoding either *Ngb* or *Aifm1* into each cerebellar hemisphere of Hq mice significantly improved mitochondrial function, including ETS and TCA activities, and neurodegeneration [88]. Importantly, both AAV-treated groups demonstrated significant improvement in motor coordination and cognitive performance, highlighting the therapeutic potential of cerebellar-targeted AAV2/9 gene delivery in mitochondrial-related neurodegeneration [88]. Notably, *Ngb* overexpression appeared to compensate for AIFM1 deficiency, by an unknown mechanism. This strategy thus offers a potential platform for treating a wide range of neurodegenerative disorders beyond those caused by a single genetic mutation, but independent confirmation is highly needed.

Friedreich’s ataxia (FRDA) is a multisystem mitochondrial disorder caused by GAA repeat expansions in the FXN gene, reducing frataxin expression and impairing iron–sulfur cluster biosynthesis [89]. AAV-mediated FXN gene replacement has demonstrated strong efficacy in multiple conditional knockout (CKO) mouse models [90]. In a skeletal muscle and heart-specific *Fxn*^*-/-*^ (Mck-CKO) model, systemic administration of a AAVrh10-CAG-h*FXN* vector prevented cardiomyopathy and premature death when given pre-symptomatically, and restored cardiac function, reduced fibrosis, and extended survival when administered after the disease onset [91]. However, supraphysiological FXN overexpression caused muscle atrophy and kyphosis in the same mouse model, emphasizing the need for dose control and tissue specificity [92].

In mouse models lacking *Fxn* in proprioceptive neurons or neuron-specific enolase expressing cells (Pvalb-CKO, Nse-CKO), AAV9- and AAVrh10-h*FXN* improved motor function, reduced neurodegeneration, and prolonged survival. Pre- and post-symptomatic treatments enhanced coordination and neuronal preservation, though some atypical phenotypes (e.g., tremors, seizures) persisted [93, 94].

Collectively, these findings indicate that *FXN* gene augmentation is a promising approach for both cardiac and neurological symptoms in FRDA. Accordingly, the AAVrh10-*hFXN* vector is advancing toward clinical trials (NCT05302271; NCT05445323).

The administration of wild-type PHP.B-*Fdxr* cDNA in a mouse model of lethal mitochondrial neuropathy with optic atrophy [95], carrying the p.Arg389Gln mutation led to robust transduction of both central and peripheral tissues [96].Treated mice showed a significant improvement in OXPHOS complexes function, sciatic nerve conductivity, and mobility. The therapy also prevented optic atrophy and reduced signs of sensory neuropathy, neurodegeneration, and chronic neuroinflammation in the brain [96].

In Barth syndrome (BTHS), caused by mutations in *TAZ* [97], encoding tafazzin, a cardiolipin modifying enzyme, AAV9-mediated gene delivery restored tafazzin expression, corrected cardiolipin remodelling defects, and improved mitochondrial structure and cardiac function. Early treatment of neonatal mice enhanced survival and prevented cardiomyopathy, while post-symptomatic therapy in older mice restored cardiac function and reduced fibrosis [98, 99].

In SLC25A46-related disorders, which impair mitochondrial fission-fusion balance and cause Leigh syndrome [100], systemic delivery of PHP.B carrying the mouse *Slc25a46* rescued survival, mobility, and mitochondrial structure in knockout pups. The treatment also corrected Purkinje cell degeneration and reduced neuroinflammation, affirming the therapeutic potential of targeting mitochondrial dynamics in CNS diseases [101].

### Gene therapy for mtDNA-Associated disorders

The multicopy nature of mtDNA, its specific genetic code and the peculiarities of mitochondrial biology, which relies on specialized machineries to import proteins across the outer and inner mitochondrial membranes, pose particular challenges for the development of gene therapy approaches for mtDNA-related disorders [1, 102].

**Fig. 3:**
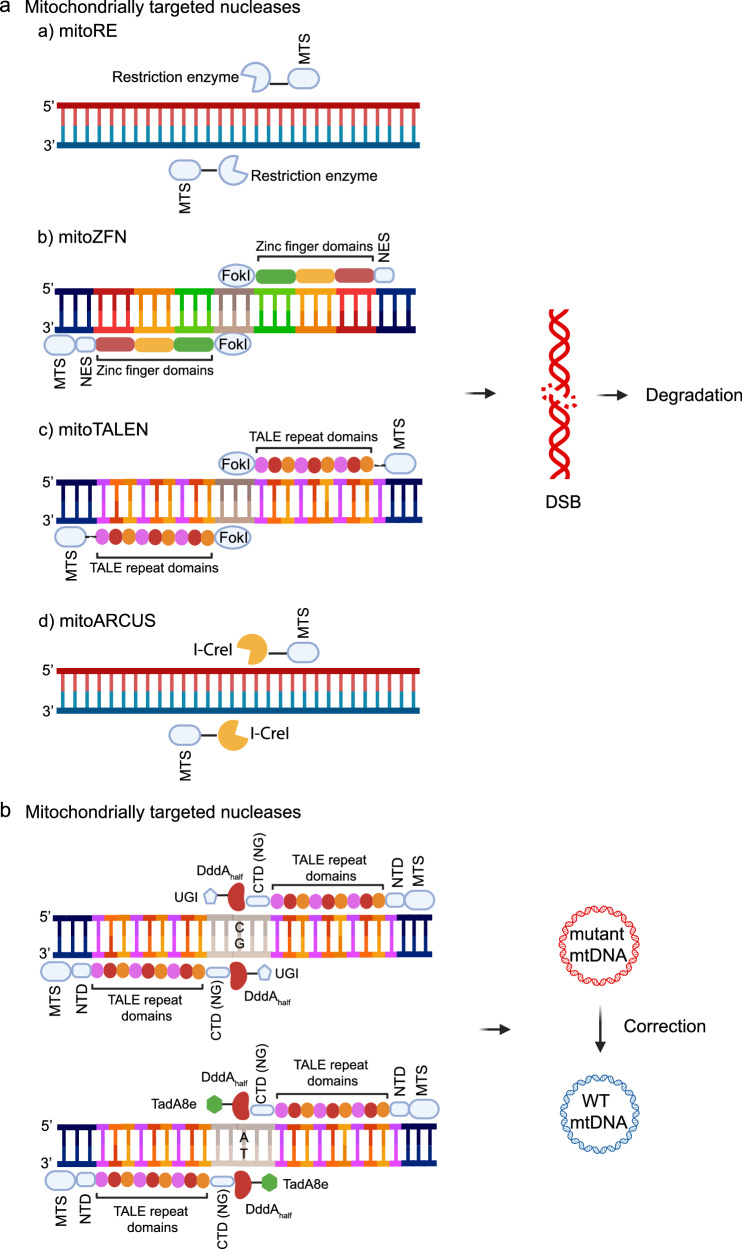
Editing tools for mtDNA. Mitochondrial nuclease and b) mitochondrial base editors. The figure illustrates the architecture of the mitochondrial gene editing platforms described in this review. All these proteins use a mitochondrial targeted sequence to reach the mitochondrial matrix and bind to the mtDNA. MTS: mitochondrial targeted sequence; NES: nuclear export signal, CTD (NG) C-terminal domain with NG repeat variable diresidues, NTD N-terminal domain; I-CreI: intron-located homing endonuclease from *Chlamidomonas reinhardtii*; UGI: Uracil Glycosylase Inhibitor; DddA_half_: double-stranded DNA-specific cytidine deaminase (half refers to the fact that it is split in two halves); CTD (NG): C-terminal Domain (Next Generation) variants of Cytosine Base Editors; TadA8e: TALE-binding protein deaminases. Figure prepared with Biorender.

Two main adeno-associated virus (AAV)-based strategies have been proposed to address mtDNA mutations. The first seeks to reduce mtDNA heteroplasmy by selectively eliminating or editing mutant genomes. Importantly, the editing approach can also target homoplasmic mutations (Fig. 3). Key challenges include achieving efficient editing across a sufficient number of mtDNA copies and delivering the tools to an adequate number of affected cells, especially within the central nervous system. Nevertheless, mitochondria-targeted genome editing technologies, such as mitoTALENs and DdCBEs, have demonstrated promising potential to selectively eliminate or correct mutant mtDNA, at least in peripheral tissues [103, 104].

**Fig. 4:**
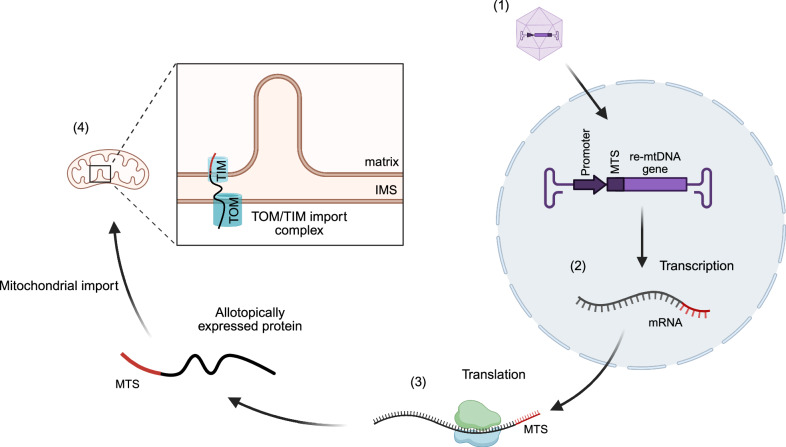
Allotopic expression and mitochondrial import of recoded proteins. A mitochondrial gene is recoded for nuclear expression and designed for mitochondrial targeting. After nuclear gene delivery (**1**), the vector directs the transcription of the mitochondrially targeted gene (**2**), which is then translated into protein by cytosolic ribosomes (**3**). The protein is subsequently transported to the mitochondria (**4**). Upon reaching the mitochondria, the precursor protein is imported via the TOM and TIM complexes. Figure prepared with Biorender.

The second approach builds on the possibility to express the wild-type form of the mutated gene from the nucleus, a process called allotopic expression [105] (Fig. 4).

#### Elimination of mutant mtDNA by using mitochondrially targeted nucleases

Mitochondrial-targeted restriction endonucleases (mitoREs), created by fusing mitochondrial targeting sequences to nucleases like mitoPstI, mitoSmaI and mitoApaLI, were early tools to induce double-strand breaks (DSBs) in mtDNA and promote heteroplasmy shift [103, 106]. In particular, the m.8993 T > G mutation associated to neuropathy ataxia retinitis pigmentosa (NARP) syndrome creates a SmaI/XmaI site absent in wild type. Delivery of mito-SmaI/mitoXmaI to cybrids with >90% mutant mtDNA substantially reduced heteroplamy [107].

To overcome the limitations of mitoREs, programmable nucleases such as mitochondrial zinc finger nucleases (mtZFNs) and transcription activator-like effector nucleases (mitoTALENs) have been developed. These are used to target the unspecific FokI endonuclease to specific loci in mtDNA to induce double strand breaks and trigger the degradation of mutant mtDNA. Both mtZFNs and mitoTALENs have shown promising results in both cellular models and the m.5024 C > T tRNAAla mouse model, by selectively eliminating mutant mtDNA and restoring mitochondrial function in cardiac tissue [108–110]. However, they face limitations in editing efficiency, design complexity, and potential off-target effects.

MitoTALENs have also been successfully used in induced pluripotent stem cells harbouring the m.3243 A > G MELAS mutation, restoring normal mitochondrial respiration and energy metabolism upon editing [111].

AAV9-mitoTALENs, specific for the m.5024 C > T mutation, was delivered by intramuscular, intravenous, and intraperitoneal injections in tRNAAla mouse model, and resulted in efficient transduction of both skeletal muscle and heart tissues. The treatment led to a significant and stable reduction of mutant mtDNA levels, and restoration of the tRNAAla levels. To selectively eliminate mutant mtDNA in the CNS of the same murine model, the mitoTALENs were delivered by using an AAV-PHP.eB vector along with a neuron-specific synapsin promoter. The treatment resulted in significant transduction across various CNS regions and a marked reduction in mutant mtDNA. Additionally, mitochondrial tRNA alanine levels, which are severely depleted by the m.5024 C > T mutation, were restored [112].

These findings show that mitoTALENs effectively reverse disease phenotypes in mice, supporting their potential as a therapeutic approach for mitochondrial disorders. However, mitoTALENs also face significant delivery challenges due to their large size, which complicates packaging into viral vectors like AAVs.

The mitochondria-targeted meganucleases (mitoARCUS) are possibly the most promising gene-editing tool for mtDNA [113]. MitoARCUS is based on the I-CreI homing endonuclease, which consists of monomers capable of inducing precise double-strand breaks at any sequence-specific sites[114, 115]. Recent studies using AAV9 vectors to deliver mitoARCUS to skeletal muscle, heart, and liver successfully eliminated the pathogenic m.5024 C > T mt-tRNAAla mutation, restoring normal tRNA levels [113]. In a mouse xenograft model, the systemic delivery of mitoARCUS targeting the common pathogenic m.3243 A > G mutation via AAV9 led to a dose-dependent reduction in mutant mtDNA within tumour tissues, with statistically significant heteroplasmy shifts observed at the highest viral dose [116]. Their relatively small size (~ 1.1 kb) and long recognition sequences (~ 22 bp) confer high specificity, making them attractive for clinical applications.

However, such approaches are uniquely applicable to heteroplasmic conditions, where reducing the proportion of mutant genomes below a pathogenic threshold can result in meaningful phenotypic rescue. Nonetheless, when using programmable nucleases like mtZFNs or mitoTALENs, careful consideration must be given to the transient loss of total mitochondrial DNA, since selective degradation of mutant genomes can precede the compensatory replication of wild-type mtDNA [109, 117].

#### Editing of mtDNA mutations using base editors

While programmable nucleases have proven useful in shifting heteroplasmy by triggering degradation of mutant mtDNA, they are unable to edit mtDNA variants. The CRISPR-Cas9 system is an obvious candidate to this end, but it is currently not applicable to edit the mitochondrial genome due to the absence of an effective import of nucleic acids, including guide RNAs, into this organelle [40], despite some successful reports.

New gRNA-free editing tools are being developed at an incredibly fast pace [118]. Double-stranded DNA (dsDNA) cytosine deaminase (DddA)-derived cytosine base editor (DdCBE) is a RNA-free method capable of correcting mutated cytosines to uracil and then thymidine, leading to C:G to T:A transitions. A critical component of DdCBE is the Uracil DNA Glycosylase Inhibitor (UGI), which prevents uracil excision from DNA.

DdCBEs have been used to generate mouse models harbouring mtDNA mutations, a critical step for understanding the inheritance of mtDNA mutations, the pathogenesis of mtDNA-related diseases and the development of new therapies. DdCBEs delivered to the mouse heart using liver detargeted AAV9.45 introduced the expected edit in *MT-ND3*, with the majority of changes on the target cytosine and minor editing in the surrounding cytosines within the editing window [119]. Similarly, DdCBEs were used to introduce several mutations in *MT-ND5*, including the m.12918 G > A mutation, corresponding to the 13513 G > A mutation in patients with variable clinical syndromes, including Leigh disease, LHON and MELAS [120].

DdCBEs were also used as a gene therapy tool in the mt-tRNAAla mouse model [121]. As directly correcting the m.5024 mutation was technically challenging, DdCBEs were used to introduce a second, compensatory mutation at position m.5081 C → T, restoring the proper base pairing in the tRNA stem. DdCBE was delivered to mice using AAV9 and resulted in the normalization of mt-tRNAAla levels and improved mitochondrial function [121].

Modifications to DdCBEs design by replacing alanine for amino acid residues at the interface between the split DddAtox halves led to the development of high-fidelity DdCBEs (HiFi-DdCBEs), which have enhanced editing efficiency while minimizing off-target effects [122].

HiFi-DdCBE was successfully used to generate mice harbouring the pathogenetic G11778A mutation in *MT-ND4*, the most common LHON variant [123]. The mice showed retinal ganglion cell (RGCs) loss and impaired visual function, despite low heteroplasmy levels. However, heteroplasmy was not directly tested in RGCs. The same paper also reported successful correction of the mutation by intravitreal injection of an AAV encoding TALE-linked deaminases (TALEDs) with minimal off-target effects. These data highlight the potential of editing tools to both generate new models of mtDNA-related diseases and their correction.

As mentioned above, DdCBEs can only convert cytosine to thymine (C-to-T) in mtDNA, which restricts their ability to target about 10% of all known pathogenic mtDNA point mutations. To expand the range of treatable mutations, adenine base editors have been designed to perform A-to-G conversions. These tools combine TALE proteins with engineered adenine deaminases and modified DddA components, enabling precise A:T to G:C changes. Potentially, this approach could correct about 43% of disease-causing mtDNA mutations, including those linked to LHON, MELAS, and Leigh syndrome, making it a significant step forward in gene therapy for mtDNA-related diseases [124]. To evaluate the effectiveness of adenine base editors on mt-Atp6 in vivo, Mutti and colleagues delivered them to neonatal mice using AAV9 vectors. Despite successful editing in cells, the in vivo efficiency and specificity within the editing window were extremely low, and only minimal changes were seen in heart tissue and none in other organs, highlighting the current limitations of adenine base editors [125].

Besides DdCBEs, other base editors for mtDNA have emerged, which combine mitochondria-targeted TALE-binding protein with either deaminases like TadA8e (for A-to-G editing) or ABOBEC1 (for C-to-T editing), enabling efficient and specific base editing [126]. These mitoBEs have shown promise in patient-derived cells, particularly for diseases like LHON, where edited cells exhibited increased ATP production and enhanced respiration, further supporting their therapeutic potential [126].

Recent advancements in mitochondrial base editors (mitoBEs) have primarily focused on introducing mtDNA mutations, with only one instance of correcting a pathological mutation [127, 128]. However, engineered mitochondrial adenine base editors (eTd-mtABEs), achieved up to 87% editing efficiency in human cells with reduced off-target activity [129]. By replacing DddA with DNA nickases, eTd-mtABEs showed a 3.2-fold improvement in strand-selective A-to-G editing over earlier versions. This system successfully generated rat models for sensorineural hearing loss (up to 44% targeted mutations) and Leigh syndrome (up to 74% heteroplasmy). Crucially, this platform also corrected mtDNA mutations, restoring wild-type alleles to approximately 53% via C-to-T base editing, which ameliorated the disease phenotype.

Despite these breakthroughs, challenges for clinical translation persist. These include sequence context limitations (e.g., 5′-TC motif in DdCBEs), varying tissue-specific efficiencies (particularly for adenine editors), and ongoing concerns about mitochondrial off-target edits. Furthermore, the large size of TALE-based systems complicates viral delivery, and even smaller alternatives like ZF-DdCBEs carry safety risks. Continued optimization is essential to improve specificity, efficiency, and delivery for therapeutic use.

#### Allotopic expression

Allotopic expression, ie the expression of mtDNA-encoded proteins from the nucleus, involves recoding mitochondrial genes to follow the universal genetic code, expressing them in the nuclear-cytosolic compartment, and adding mitochondrial targeting sequences (MTS) to direct the proteins to mitochondria [105, 130]. Although conceptually straightforward, this method faces major challenges, particularly due to the hydrophobic nature of mtDNA-encoded proteins and the lack of chaperones needed for proper import and folding. Consequently, solid evidence for successful mitochondrial import and functional integration of allotopically expressed proteins into OXPHOS complexes remains limited.

Preclinical studies targeting mtDNA-encoded genes like *ND4*, *ND6*, *COX2*, and *ATP6* have shown some progress. For example, allotopic expression of human *ND4* in wild-type mice was safely delivered to mitochondria [131], and restored visual function and preserved retinal structure in a LHON mouse model [132]. Similarly, in a rat model, ocular delivery of AAV carrying human *ND4* achieved mitochondrial import, Complex I assembly, and preserved electron transport system activity, preventing retinal ganglion cell degeneration [133].

Allotopic expression presents several conceptual hurdles. First, it was shown that apparent functional rescue after allotopic expression of mt*-ND6* in homoplasmic cybrids was actually due to revertant mtDNA clones rather than true mitochondrial import and integration. Highly hydrophobic proteins such as ND4, ND6, and cytochrome b show poor mitochondrial import efficiency and often misfold or aggregate in the cytosol, causing proteotoxic stress or degradation. Even when imported, proper assembly into multimeric OXPHOS complexes is difficult, as mismatched expression or folding kinetics impair complex stability [105, 134, 135].

Second most of the experiments used the human gene in rodent models, which goes against very solid evidence of poor inter-species compatibility of mitochondrial proteins [130, 131].

Despite the limitations and technical challenges associated with allotopic expression, this strategy has progressed into clinical evaluation for LHON. Gene therapy approaches targeting the m.11778 G > A mutation using a recombinant adeno-associated virus vector (rAAV2/2-*ND4*) have been developed by several groups. Four phase III studies—RESCUE, REVERSE, RESTORE, and REFLECT—have evaluated the efficacy and safety of lenadogene nolparvovec (GS010/LUMEVOQ®).

In the RESCUE (NCT02652767) [136] and REVERSE (NCT02652780) [137] trials, a single intravitreal injection of lenadogene nolparvovec (9 × 10¹⁰ viral genomes/eye) was administered to patients with subacute (RESCUE) or dynamic (REVERSE) LHON. Neither study provided definitive evidence of a unilateral therapeutic effect, failing to meet the primary endpoint between treated and sham-treated eyes at 48 weeks. Instead, both treated and sham eyes demonstrated similar visual improvement, resulting in minimal between-eye differences. Participants were then invited to participate in an extension study for a follow-up of five years after treatment (RESTORE; NCT03406104). The treatment effect of lenadogene nolparvovec on BCVA (Best-Corrected Visual Acuity) and vision-related quality of life was sustained at 3 years, with a maximum follow-up of 52 months (4.3 years) after the onset of vision loss [138].

The REFLECT (NCT03293524) trial similarly failed to demonstrate a clear treatment effect. Although all participants received gene therapy in the first affected eye, randomisation of the second eye to active treatment or placebo did not produce a meaningful difference in visual outcomes.

However, although modest, these outcomes differ significantly from the published natural history of LHON.

## Conclusions and future perspectives

The rapid evolution of AAV–mediated gene therapy has significantly expanded the therapeutic landscape for genetic diseases, including mitochondrial disorders. However, significant challenges still limit the use of AAVs, including limitations in manufacturing, administration route, immune response to the transgene and/or capsid proteins, limited cloning capacity.

The manufacturing costs of clinical grade AAVs are extremely high, partly due to the complex regulatory requirements [139]. This is particularly challenging for mitochondrial diseases, which can be caused by mutations in over 400 genes, making it unaffordable the development of gene therapies for every and each of them. Streamlining the AAV production and safety testing, simplifying the regulations, and creating public-private alliances may only partly solve this issue [139].

While preclinical work shows that peripheral tissues are relatively easy targets for AAVs, significant challenges are still present for the brain, often affected in mitochondrial diseases. Several routes have been exploited to allow AAVs to cross the blood-brain barrier (BBB). These include the intranasal, intrathecal, intraparenchymal, intracerebroventricular and intra-cisterna magna routes [140]. However, there is a strong competition to generate new AAV-serotypes with increased capacity to cross the BBB, as this would entail the possibility of intravascular deliver, which would be by far less invasive than other routes [141–144].

Ongoing clinical trials for other genetic diseases unravelled different sources of toxicity in AAV-based therapies. These include hepatotoxicity, dorsal root ganglion toxicity, thrombotic microangiopathy and cardiotoxicity. Adverse effects of AAV-based gene therapy are now well-documented [145] and led to the suspension of some clinical trials, as a consequence of the death of some patients [146]. Several factors may contribute to toxicity, such as pre-existing antibodies against AAVs, the high doses often required to achieve therapeutic effect, leading to massive presence of capsid proteins and/or transgene. Improving manufacturing and developing new, more effective serotypes may address this issue, reducing the need for very high titres. In addition, the recent identification of an alternative entry pathway for some AAVs, relying on a type I transmembrane protein carboxypeptidase D (AAVR2), suggests that a better understanding of AAV-host interaction may identify more specific targets, which may be exploited to reduce the viral dose [147].

Finally, the limitations of AAV cloning capacity can be overcome by using split vectors, which divide the therapeutic gene on several plasmids. The complete gene is then reconstituted by either exploiting the splicing machinery or the inteins domains which allow recombination at the protein level [148, 149].

Despite these difficulties AAV-based gene therapy is still the most robust, and overall safe platform to deliver therapeutic genes in different genetic conditions, including mitochondrial diseases.
